# Structural determinants in quality of care for older adults: a nursing perspective on medical services - qualitative findings from focus group discussions in Germany

**DOI:** 10.1186/s12912-026-05133-6

**Published:** 2026-07-29

**Authors:** Evelyn Kleinert, Laura Mohacsi, Eva Hummers

**Affiliations:** Department of General Practice, University Medical Center Göttingen, Humboldtallee 38, 37073 Göttingen, Germany

**Keywords:** Medical progress, Moral distress, Overtreatment, Staffing shortages, Person-centered care, Older adults, End-of-life care, Nursing

## Abstract

**Background:**

Aging populations, multimorbidity, and rapid medical progress have expanded treatment options in old age, but also intensified ethical and organizational challenges in everyday care. Little is known about how nurses, as key frontline professionals, experience these structural developments specifically in the context of medical and nursing care for older adults in Germany. Therefore, this study aimed to examine how nursing staff perceive structural conditions in the German healthcare system and their effects on medical and nursing care for older patients.

**Methods:**

This study employed a qualitative analysis of five focus group discussions with 28 nurses from hospitals, nursing homes, and home-based nursing services in Germany. Participants with diverse qualifications and work settings were purposively and conveniently sampled, and discussions were stimulated by case vignettes on elective surgery and life-prolonging interventions in very old age. Data were analyzed using Kuckartz’s Qualitative Content Analysis.

**Results:**

Nurses described medical progress as simultaneously enabling better symptom control, mobility, and end-of-life care, while also prolonging phases of frail survival with high care dependence. They reported strong treatment pressures and financially driven incentive structures that foster overtreatment and can conflict with older patients’ wishes and perceived quality of life. Chronic staff shortages and time constraints led to “shortcuts” such as tube feeding or increased use of psychotropic medication, which were experienced as undermining person-centered care and professional ethics. Across settings, nurses highlighted a loss of professional influence in medical decision-making, feelings of powerlessness, and moral distress linked to implementing decisions they had not shaped but were responsible for carrying out.

**Conclusions:**

From a nursing perspective, good medical care in old age depends not only on technological advances and geriatric expertise but also on structural reforms of financing, staffing, and decision-making processes. Strengthening nurses’ autonomy, involvement in treatment decisions, and ethical support within interprofessional teams may help align medical interventions more closely with older people’s concepts of a good life and a good death.

**German clinical trials register:**

DRKS00027076, 05/11/2021. https://www.drks.de/search/de/trial/DRKS00027076/details.

**Supplementary Information:**

The online version contains supplementary material available at 10.1186/s12912-026-05133-6.

## Background

Aging populations are driving a higher share of older individuals needing medical treatment at more advanced ages. Progress in medical technology—such as minimally invasive procedures, markedly improved organ transplant outcomes, and life-sustaining therapies like hemodialysis—have made many interventions safer and more routinely available for older patients [1]. Yet this progress coincides with growing challenges in clinical decision-making for older adults, driven by rising rates of chronic diseases and multimorbidity [2]. As a result, more people now experience prolonged phases of chronic illness, heightening care complexity and creating ethical dilemmas where treating one condition risks exacerbating others [3].

To contextualize the accounts of nursing staff facing these challenges, it is essential to consider the specific framework of the German health care system. Over the past 25 years, the system has undergone profound change, shaped by a series of major reforms and financial restructuring aimed at increased efficiency and improved cost controls. A key milestone was the implementation of the diagnosis-related group (DRG) reimbursement system for hospitals in 2009, which linked hospital payments to standardized case categories rather than the length of stay or resources used. While intended to enhance transparency and cost-effectiveness, the DRG system intensified economic pressures on hospitals by incentivizing shorter inpatient stays and tighter resource management. The 2000s saw notable deteriorations in working conditions within healthcare, especially due to substantial budget cuts and cost containment measures. Both the inpatient and outpatient sectors faced additional strain due to increased patient copayments, and stricter budget limits. As a result, nursing staff encountered growing demands from new documentation requirements, more complex auditing processes, and steadily shrinking time allocations for patient care.

The hospital sector has undergone a dramatic transformation since 2002. The number of hospitals has fallen by almost 15% over the past 20 years, with numerous formerly charitable and public hospitals being acquired by private groups. Nevertheless, with 7.9 hospital beds per 1,000 residents in 2021, Germany maintains the highest bed density in the EU—50% above the average. However, the physician-to-bed and nursing staff-to-bed ratios remain low, with Germany reporting the lowest nurse-to-bed ratio in the EU [4].

Compared to other countries, Germany has a high number of doctors and nurses relative to its population. However, due to the high number of inpatients in the country, the nurse-to-patient ratio in hospitals is significantly lower than in many other countries, resulting in heavy workloads and compromised care quality (e.g. higher mortality rates, longer hospital stays and an increased risk of adverse events such as pressure ulcers, falls, pneumonia and urinary tract infections) [5]. Survey data confirm these pressures: 35% of German nurses described their ward’s quality of care as poor or fair, substantially worse than the European peer average, with only Greece reporting higher dissatisfaction [6]. A key concept for understanding nurses’ experiences in this context is moral distress, first described by Jameton (1984) as the suffering that occurs when a healthcare professional knows the ethically right course of action, but is constrained from taking it by institutional, hierarchical, or resource-related barriers. Moral distress has since been distinguished from the related concept of moral injury—a deeper and more lasting damage to one’s moral foundation resulting from repeated exposure to morally compromising situations [7]. Both phenomena have been documented among nursing staff across healthcare systems and are particularly prevalent in settings characterized by resource scarcity, high workloads, and limited professional autonomy.

German nursing education is currently undergoing major reform aimed at enhancing the professional and legal standing of nursing staff. Previously, geriatric, general, and pediatric nursing were trained separately; these are now transitioning into a unified three-year generalist nursing education, with new requirements emphasizing diverse practical experience and inter-institutional trainee exchanges. In Germany, a distinction exists between registered nurses (examinierte Pflegefachkräfte), who complete this three-year formal training program, and nursing assistants (Pflegehilfskräfte), who undergo shorter, less formalized training typically lasting one year or less. Both groups are involved in the direct care of older patients and are subject to the structural conditions described in this paper. While nursing has become a graduate profession in many countries—including the UK, Scandinavia, and North America—Germany’s academization rate remains exceptionally low by international standards. Despite ongoing academization efforts, the proportion of nurses with academic training remains at 0.8% well below the target range of 10 to 20% [8]. In international comparison, Germany pursues various special paths in the training of nursing staff and the resulting skills and areas of responsibility. This results in unattractive employment conditions with little opportunity for independent activity [9].

In response to the growing shortage of nursing staff, German health policy has pursued a dual strategy of expanding domestic training capacities and improving working conditions, while at the same time actively recruiting nurses from abroad through bilateral recruitment programs and more flexible immigration regulations. In hospitals and long-term care, foreign-trained or foreign-national nurses now represent a substantial and rising share of the workforce, with estimates suggesting that roughly one quarter of employed nurses have a migration background [10–12].

Against the backdrop of ongoing debates about structural challenges in the German healthcare system, and given that structural conditions emerged as a pervasive and recurring theme across all focus group discussions with nursing staff, we conducted a targeted secondary analysis of this data. The aim of this study was therefore to examine how nursing staff perceive changes in healthcare conditions, particularly in the context of medical care for older patients in Germany. While the original research question addressed perceptions of old age, recurring structural themes across all focus groups led us to additionally analyze the data on structural determinants as an overarching category.

## Methods

**Fig. 1:**
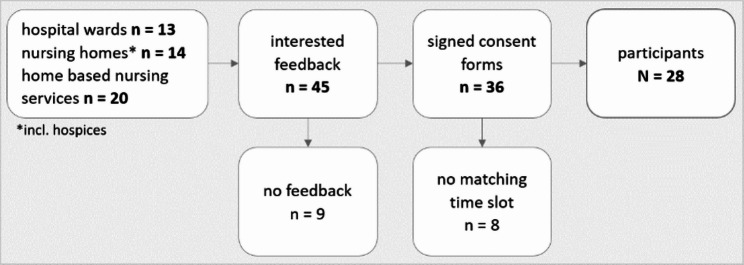
Recruitment process of participants (*N* = 28)

The present study was conducted using a qualitative research approach, with focus group discussions serving as data collection method. Between August 2022 and August 2023, two researchers (EK and LM) conducted focus groups within the facilities of the University Medical Center Göttingen. The focus group guidelines, which focus on medical care for older adults, can be found in Appendix A. Inclusion criteria required that participants were employed as nurses or nursing assistants in one of the three settings (hospital, nursing home, or home-based nursing service) and had direct experience in the care of older patients. Participants were recruited using a combination of convenience sampling and purposive sampling strategies to ensure representation from various home based and inpatient institutions. The recruitment process is shown in Fig. 1. Detailed information regarding the research design and recruitment procedures is available in the previously published study protocol [13] and in other result articles [14, 15]. Data analysis was performed using Qualitative Content Analysis according to the methodology described by Kuckartz [16]. EK and LM independently coded the transcripts and jointly developed the category system through iterative discussion. The analysis followed an inductive approach, in which the structural determinants were derived from recurring themes in the data. Sample size was guided by thematic saturation: after each focus group, EK and LM discussed the themes that had emerged, and data collection concluded once no new themes were identified. To ensure methodological rigor in reporting, the study adhered to the Consolidated Criteria for Reporting Qualitative Research (COREQ) [17]. The quotations used here were first translated from German into English by EK in an anonymized form using DeepL translation software, and were then checked again. False starts and non-lexical expressions were omitted to enhance readability and conciseness.

To ensure the data was comparable, discussions were initiated using two case vignettes (Appendix B). The development of the vignettes was a collaborative effort between EK, LM, and their physician colleagues. Utilizing real scenarios, they modified and adapted them in detail to provoke controversial discussions. The first vignette outlines a case in which the preservation of quality of life must be carefully balanced against the surgical risks: an 87-year-old woman with multiple comorbidities, including obesity, seeks the implantation of a knee endoprosthesis to alleviate arthritis-induced pain. The second vignette addresses ethical considerations surrounding life-sustaining interventions. It depicts an 81-year-old nursing home resident with advanced Parkinson’s disease and early-stage dementia, for whom the attending physicians propose the insertion of a PEG feeding tube. The development of the focus group guides and vignettes is documented in Kleinert et al. (2025) [14].

### Study context and secondary analysis

The study was conducted as part of the project “Medicine in Older Age” which is part of the research group “Medicine, Time, and the Good Life” (FOR 5022). The project examines how new medical possibilities and treatments interact with the temporal structures of human life and conceptions of a “good life”. This study originally examined the influence of perceptions of old age on medical care for old people [13–15]. Individuals over the age of 75, as well as doctors and nursing staff from various professional settings were interviewed in separate focus group discussions. Participants of all five focus groups with nursing staff consistently linked their assessments of medical care for older patients to systemic factors such as staffing shortages, financial incentives, and limited professional influence. This led us to conduct a targeted secondary analysis, systematically examining structural determinants as an overarching analytical category. The secondary analysis followed the principles of qualitative secondary analysis, drawing on existing transcripts to address a new but related research question [18]. The analysis of the structural determinants followed the same analytical procedure as the primary analysis. Approval was granted by the Medical Ethics Committee at the University Medical Center Göttingen (No. 16/9/21).

## Results

A total of 28 nursing staff members participated in the study across five focus groups. Four groups met in person, while one group met online. Each meeting lasted between 90 and 120 min. Participants’ demographic and professional characteristics are presented in Table 1.

**Table 1:** Self-reported socio-demographic and professional characteristics of participants (*N* = 28)

gender	female male	18 10
age (y)	mean (SD) range	45.8 (13.5) 25–69
qualification	registered nurse *including those with specialist training* *including those with B.A. in health and social management* registered geriatric nurse geriatric nursing assistant	22 *4* *1* 4 2
sector	nursing home hospital home based nursing service	3 17 8
professional experience (y)	mean (SD) range	17 (10.2) 1–42

**Fig. 2:**
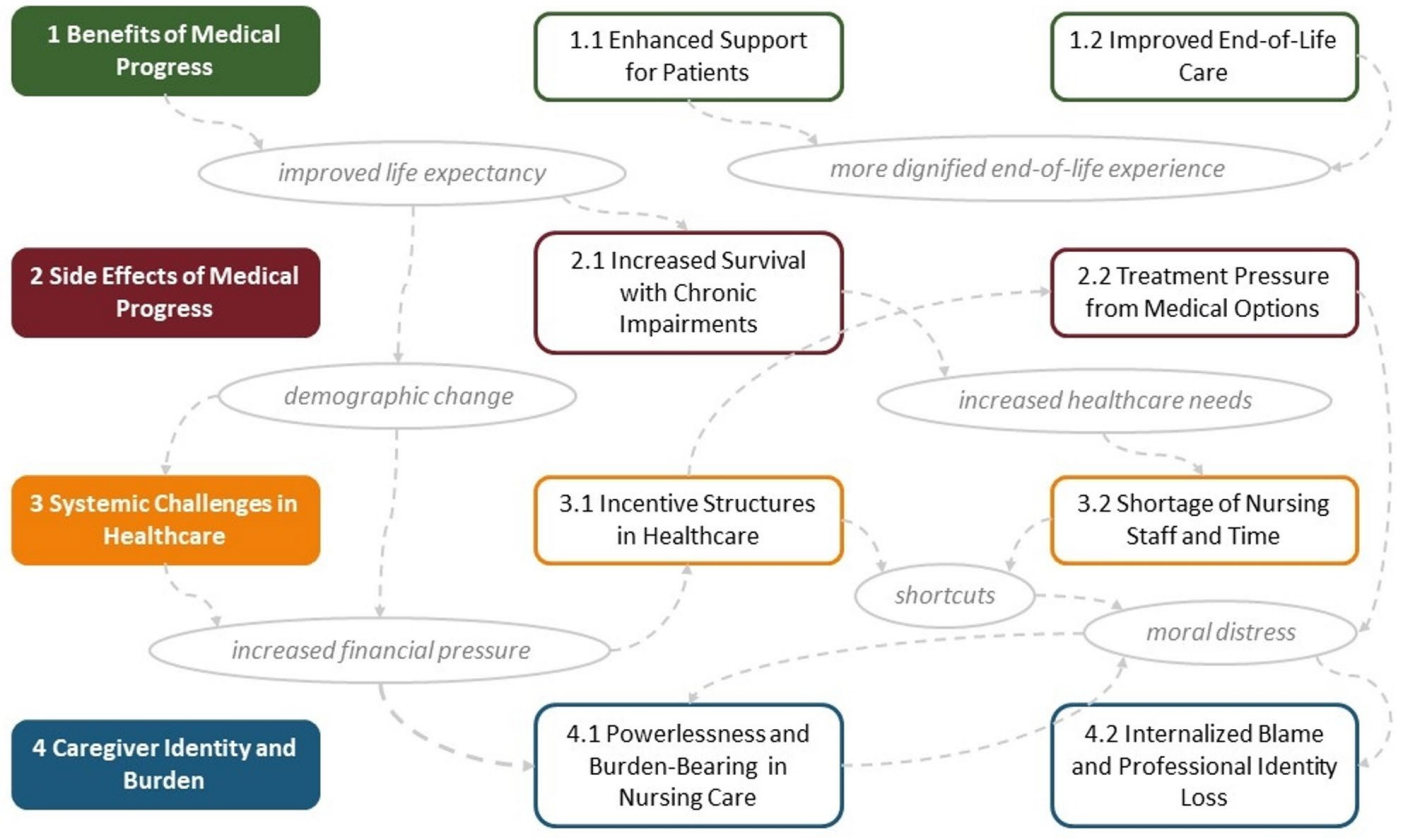
Dimensions of structural determinants

The findings are presented along four main categories: (1) benefits of medical progress, (2) side effects of medical progress, (3) systemic challenges in healthcare, and (4) caregiver identity and burden (see Fig. 2).

### Benefits of medical progress

Nursing staff across all settings acknowledged that medical progress has brought tangible benefits for older patients. The following themes highlight areas where advances have improved everyday care and quality of life.

#### Enhanced support for patients

Nursing staff observed that medical progress has significantly improved the support available to older patients, particularly through advanced medical devices and modern therapeutic options. Participants agreed that such innovations have reduced complications (such as pressure ulcers) and enhanced mobility, allowing for greater independence and quality of life, especially for those living with chronic health conditions. In particular, devices like modern wheelchairs, walkers, anti-decubitus mattresses, and overbed trapezes were seen as crucial in alleviating everyday burdens and enabling more active lives.

*What I think is very beneficial is the use of medical devices. Things like the anti-decubitus mattress*,* which didn’t exist 15 or 20 years ago. People had a lot of bedsores back then. That has decreased a lot. And all these aids*,* like wheelchairs*,* crutches*,* and walking frames. These walkers haven’t been around that long. They are a great help as a support. Also*,* these overbed trapezes to get people out of bed into wheelchairs and things like that. Yes*,* the medical devices make things much easier. (Madeleine*,* group 3*,* 55 y*,* female*,* home based nursing service)*

Overall, advancements in pharmacological treatment and assistive technology were experienced as factors that not only relieved symptoms or prevented complications, but also positively shaped patient autonomy and well-being.

#### Improved end-of-life care

Nursing staff emphasized that end-of-life care has evolved significantly, now placing much greater value on the alleviation of suffering and respect for patient autonomy. There is a consistent focus on ensuring physical comfort and dignity for patients, with increased use of palliative teams and pain specialists to manage symptoms and promote a peaceful dying process. This shift is reflected in daily practice, where interventions are chosen based on the patient’s wishes and the goal of comfort, rather than on prolonging life at any cost.

*What I have noticed is that the process of dying is better supported. For example*,* a pain specialist or someone from the palliative care team is brought in—mainly those two. … Patients don’t have to suffer anymore—they don’t have to be in terrible pain*,* they don’t have to experience anxiety*,* and they don’t have to struggle to breathe. (Manuela*,* group 1*,* 49y*,* female*,* hospital care)*

Participants described how advance directives are increasingly accepted and honored, and how the willingness to refrain from life-prolonging measures has grown. They also noted persistent organizational and legal hurdles, but observed that respect for patient preferences at the end of life has become more evident, even in acute emergency settings.

### Side effects of medical progress

At the same time, participants identified significant unintended consequences of medical progress. The following themes reflect the ambivalence nursing staff feel toward technological and medical advances in the care of older adults.

#### Increased survival with chronic impairments

Nursing staff consistently observed that medical progress has prolonged the lives of many older people, including those with serious conditions that would have been fatal in the past. However, this increased longevity often comes with severe chronic impairments—such as frailty, sensory loss, or multiple comorbidities—that can significantly diminish quality of life. Many participants expressed concern about the growing number of older patients living with profound limitations who nevertheless require continuous, resource-intensive care, often in settings ill-equipped for such complex needs.

*We’re creating patients who have to be cared for somewhere*,* but in the end*,* nobody really wants that anymore*,* because there just isn’t enough staff for it. Because it’s expensive*,* because of so many reasons. (Hossam*,* group 1*,* 63y*,* male*,* hospital care)*

While extending life is a major achievement of modern medicine, nursing staff highlighted the need for greater attention to the lived experience, support, and social participation of individuals who now survive for years with substantial health burdens.

#### Treatment pressure from medical options

Nursing staff noted that the continuous expansion of medical possibilities has led to a subtle yet powerful expectation to exhaust all available treatments—even when interventions may not align with the patient’s wishes or best interests. The ease of access to sophisticated technology and advanced therapies can inadvertently encourage overtreatment, fueled by organizational routines and the desire to avoid blame. Nurses described how families and staff alike sometimes experience pressure to “do everything possible” simply because options exist, even in cases when restraint might be more appropriate. At the same time, nursing professionals emphasized that physicians often experience this treatment pressure even more acutely, as they bear ultimate responsibility for clinical decisions and may fear legal consequences if interventions are withheld. This dynamic reinforces the authority of doctors in such situations, making it difficult for nurses and relatives to raise objections, even when patient autonomy or quality of life might argue for a more conservative approach.

While most accounts reflected professional nursing experience, some participants also drew on personal experiences as family members. These two perspectives were not always clearly separable, as personal encounters with the healthcare system as a relative repeatedly informed and shaped participants’ professional assessments of care situations. The following statement illustrates how personal experiences with pressure to provide treatment can be intertwined with professional perspectives in nursing:

*My mother was in the ICU at the university hospital for weeks*,* she was intubated. She had a brain hemorrhage*,* and then she got a real case of the flu. Her fever went up to 41°C*,* and whenever she had a lucid moment*,* because of the fever*,* she made it clear that she didn’t want to go on anymore. I was there with a doctor and a nurse*,* and she made that very clear to me. In the end*,* I was called in for a meeting*,* and there were four people sitting around me. ‘People don’t die from the flu anymore these days’*,* they said. So*,* they convinced me to agree to a tracheostomy. And in the end*,* how much longer did she live? Two and a half weeks. (Birgit*,* group 2*,* 56y*,* female*,* hospital care)*

These accounts describe an important ethical dilemma: while progress in medicine provides more treatment options, they can also generate unintended pressure to act, sometimes overshadowing patient autonomy and considerations of quality of life.

### Systemic challenges in healthcare

Nursing staff described a number of persistent systemic barriers in the provision of care for older adults. Key issues include incentive structures that prioritize finances over patient needs, chronic staff shortages resulting in time constraints, and an erosion of professional identity and influence. These factors shape both day-to-day practice and broader patterns of care, sometimes to the detriment of older patients’ well-being.

#### Incentive structures in healthcare

Nursing staff described incentive structures as a persistent and cross-sectoral challenge in Germany’s healthcare system. Financial motives, quotas, and contractual obligations—rather than patients’ needs—often shape clinical decisions. In particular, nurses with work experience in hospitals reported that interventions are frequently guided by the prospect of revenue or the requirements of institutional contracts, especially in surgical disciplines and where hospitals collaborate with private practitioners. This focus on throughput and financial performance was seen as driving overtreatment, with decisions sometimes being justified using paternalistic arguments or institutional goals, often at odds with patient welfare.

*Yeah*,* it’s just about the money now. It’s not about the person anymore. … And then the chief physician sits there and says*,* ‘Well*,* if this were my mother*,* I would do it*,*’ and you’re thinking to yourself*,* ‘Man*,* she’s just terminally ill. We really don’t need to do this surgery.’ … Every day definitely brings in money. That’s the problem. (Birgit and Robert*,* group 2*,* 56y*,* female and 54y*,* male*,* both hospital care)*

Another participant drew attention to the specific influence of private orthopedic contractors in hospitals:

*Now you really have to take a look at the system. Who’s actually doing these surgeries? It’s private orthopedic surgeons who have admitting privileges at hospitals. And they make fixed contracts—they commit to doing*,* say*,* 200 hip replacements and 150 knee replacements per year*,* and they have to meet those numbers. (Steffen*,* group 5*,* 53y*,* male*,* hospital care)*

These practices were seen as resulting in unnecessary surgeries, illustrating a technical imperative—the tendency to use available medical technologies and procedures simply because they exist and can be applied—while giving diminished weight to patient needs or wishes. Research interests, ambition for clinical experience, and job security are further contributing factors. Nursing staff were concerned that these conflicting incentives undermine the integrity of care for older people, contributing to a system in which patient-centered ideals are repeatedly compromised.

#### Shortage of nursing staff and time

A pervasive theme in the focus groups was the critical shortage of qualified nursing staff, which severely limits the time available to care for older patients. Nurses across settings described how this scarcity robs patients of meaningful interaction and personalized care, often reducing complex needs to routine tasks or medication administration. This lack of time diminishes the quality of care and leads to ethical tensions. For example, clinical shortcuts are used to compensate for staff shortages, such as the use of psychotropic drugs to reduce the emotional distress due to loneliness or tube feeding instead of time-consuming oral feeding.

*It truly is a problem for everyone*,* because all the nursing staff simply don’t have any time. But people who are older*,* sick*,* or in need of care—they need time*,* and they also need someone to talk to. I think a lot of older people are alone*,* especially because of what’s always diagnosed as age-related depression. … All the nursing staff just don’t have any time anymore*,* and I think you could say the same for the doctors. (Gabi*,* group 3*,* 28y*,* female*,* home based nursing service)*

*Of course*,* you have to remember*,* he lives in a nursing home. And now*,* as I’ve been thinking about it*,* the staff shortage—it’s obviously easier for the nurses in the nursing home to say*,* ‘Oh*,* just go ahead and do it!’ [referring to feeding tube insertion]. It’s easier for them. They hook up the food*,* inject it in*,* and boom*,* done. I don’t have to stand there forever and feed him thickened food. That takes time*,* too. I don’t have that. I just don’t have the time. (Melissa*,* group 4*,* 32y*,* female*,* home based nursing service)*

Participants noted that facilities are increasingly recruiting nurses from abroad to address chronic staff shortages. While they acknowledged the professional competence of internationally trained colleagues, they also reported concerns about language barriers causing significant communication difficulties, particularly when caring for older patients with complex needs, cognitive impairment, or hearing difficulties.

Nurses also reported psychological distress and burnout resulting from chronic understaffing and overwhelming workloads, compounded by a sense of powerlessness when unable to provide care that meets their own professional standards.

*It’s often a mix of understaffing*,* frustration*,* and burnout. Because*,* if you think about it*,* if you have to work like that every day—if I*,* as a nursing assistant*,* have to care for 30 people every day—then I definitely lack the knowledge and time to provide proper care for someone like that… You just can’t manage it. No matter how dedicated or skilled you are*,* it’s impossible to do justice to everyone. (Oliver*,* group 4*,* 31y*,* male*,* hospital care and home care*,* temporary staffing agency)*

Participants across all settings described how these compounding pressures left them unable to meet their own professional standards of care, contributing to a pervasive sense of ethical compromise in daily practice.

### Caregiver identity and burden

Beyond systemic and financial barriers, nursing staff described how everyday care work shapes their professional identity, often resulting in experiences of powerlessness and emotional burden.

#### Powerlessness and burden-bearing in nursing care

Nursing staff reported a deep sense of powerlessness in daily practice, especially when major care decisions are made by physicians or family members without meaningful input from those providing day-to-day support. This lack of agency can leave nurses carrying the emotional and practical burden of implementing and managing the consequences of choices for which they feel little ownership.

*But unfortunately*,* it’s often the doctors on the ward who make that decision*,* or sometimes the family members. … And then after the surgery*,* the patient was clear-headed enough to understand what had happened and said*,* I never even wanted this. And that’s when the trouble starts. Then they end up lying in a nursing home*,* complaining for two years because they didn’t want this*,* constantly pulling out the tube or tearing something off. … So you come in and everything is on the floor*,* tube feeding everywhere. … And then you have to clean up the mess*,* or in the end*,* you might even get blamed for it. (Oliver*,* group 4*,* 31y*,* male*,* hospital and home care*,* temporary staffing agency)*

Institutional protocols often further reinforce this sense of helplessness, obligating nurses to follow procedures or persist with interventions even in situations that feel futile or ethically questionable.

*We have to perform resuscitation until the doctor arrives. Why? Well*,* why? Because we never find a person who is dead—we find a person with no detectable vital signs. And that’s the definition until an emergency doctor arrives*,* or maybe the family doctor happens to walk by and says*,* ‘This person is dead.’ Until then*,* we’re not allowed to lock the door or say*,* ‘She passed away last night.’ (Stefanie*,* group 5*,* 31y*,* female*,* nursing home care)*

In addition to institutional constraints, caregivers — particularly those working in home-based settings — must deal with the personal preferences of patients and their families. If a caregiver’s personal characteristics — such as their gender or ethnicity — are disliked, management needs to send someone else.

*If a patient doesn’t like us for some reason—maybe they don’t like the way we look or something—then it’s*,* “No*,* we don’t want them anymore.” Or*,* for example*,* we have a colleague with darker skin. Yes*,* some patients have refused to be cared for by him because of his darker skin color. And others love him. That’s just how it is. It’s always so difficult. (Elke*,* group 2*,* 58y*,* female*,* nursing home care)*

This does not only undermine the caregiver’s professional identity but also exposes them to discrimination and further emotional strain. They must tolerate not only the burdens imposed by hierarchical healthcare structures and rigid protocols but also the unpredictable and sometimes prejudiced demands of those they serve. Within this complex environment, the traditional nursing role as an advocate for those with need for care becomes increasingly difficult to uphold: the moral obligation to speak for people’s needs and wishes often conflicts with institutional authority, procedural compliance, and resource constraints, leaving caregivers torn between professional ethics and systemic limitations.

#### Internalized blame and professional identity loss

Participants contrast the situation in Germany with the one perceived in other countries, where nurses and doctors are believed to work more closely together. They argue that there has been a long-term trend in Germany to separate nursing and medical tasks. Some of them attribute this to their own colleagues, who repeatedly refuse to handle certain tasks, arguing that they are medical duties.

*I know the discussion—it’s always*,* “That’s not our job*,* that’s a doctor’s job.” We don’t change dressings anymore*,* we don’t draw blood anymore*,* we don’t do this or that anymore. And of course*,* we’ve ruined our own career prospects because of it. (Steffen*,* group 5*,* 53y*,* male*,* hospital care)*

Nursing staff face a professional dilemma due to ever-increasing workloads. One strategy is to refuse tasks that are technically medical activities, which are therefore the responsibility of doctors, but commonly delegated to nursing staff. Some participants criticized their own profession for distancing from core clinical responsibilities, which they argue has contributed to a loss of status and involvement in medical care. Refusing to perform tasks like blood draws or dressing changes, they cautioned, risks narrowing nursing’s role and weakening its professional identity. In this context, the advocacy dilemma reappears—nurses who withdraw from direct medical engagement may protect themselves from overburden but simultaneously lose the proximity and influence needed to effectively advocate for their patients’ interests.

## Discussion

The findings of this study illustrate how medical progress, structural transformations in the German healthcare system, and the specific positioning of nursing as a profession jointly shape the possibilities and limits of good medical care in old age from the perspective of nursing staff. Nurses perceive medical progress simultaneously as an expansion of possibilities for autonomy and quality of life in old age (assistive devices, palliative care) and as a source of prolonged phases of frail survival characterized by multimorbidity and a high need for care. This prolonged phase of frail survival aligns with Gruenberg’s (1977) expansion theory (expansion of morbidity), which argues that medical progress, while extending lifespan, simultaneously increases the years lived with chronic illness and disability [19]. From this theoretical perspective, the nurses’ observations are not incidental but structurally anticipated: a healthcare system that successfully keeps more people alive longer will inevitably generate a growing population of older adults with complex, resource-intensive care needs. What our findings add to this theoretical framework is the nurses’ perspective on how this expansion of need collides with a system not structurally equipped to meet those needs—in terms of staffing, time, and professional support. It should be noted, however, that the compression of morbidity thesis (Fries, 1983) offers a counterpoint: advances in prevention and health promotion may compress illness into a shorter period at the end of life, increasing the years lived in good health. Empirical evidence supports both tendencies, with compression observed for conditions such as stroke and myocardial infarction, and expansion particularly evident in type 2 diabetes and multimorbidity [20]. The predominantly expansionist picture painted by the nurses in our study may thus also reflect a professional bias inherent to nursing work: by definition, nurses encounter only those who are ill, never the growing number of older adults who remain healthy and independent. This selective exposure may lead nursing staff to perceive the expansion of morbidity as more universal than population-level data would suggest.

Nurses’ descriptions of treatment pressure (“doing everything possible”) and incentive-driven overtreatment align with conceptual work on the “technological imperative” [21], which explains how scientific discoveries drive the creation of new standards of care and the broadening of health insurance benefits. According to Kaufman, these factors create an ethical obligation for doctors to offer certain procedures to older patients and for those patients to accept them. In Germany, incentive-driven treatment is especially evident in knee replacement surgery, which is performed nearly twice as often compared to the OECD average [22, p. 119]. At the same time, nurses’ narratives show how such technologically and economically driven standards of care collide with their own assessments of frail older people’s wishes and life situations, placing them in recurring ethical conflicts.

Recent work on moral distress and moral injury among healthcare workers shows that structurally generated moral stressors—such as resource scarcity, unsafe conditions and exclusion from decision-making—can accumulate over time and threaten professionals’ moral integrity. A scoping review of healthcare workers during the COVID-19 pandemic demonstrates that repeated exposure to such patient-, team- and system-level moral stressors is associated with burnout, loss of trust in institutions and intentions to leave the profession [7]. These dynamics closely mirror the experiences of nurses in our study, who describe long-standing staffing shortages, incentive-driven overtreatment and limited influence on treatment decisions in old age as sources of persistent moral strain, suggesting that moral distress is not an exceptional “crisis” phenomenon but part of everyday geriatric care. Another dimension of moral harm is the experience of discrimination against nursing staff by patients or their relatives. As the findings show, some patients refused care based on the caregiver’s skin color or gender—which places an additional and often invisible burden on the affected staff members [23]. In a healthcare system that increasingly relies on internationally recruited nursing staff to address staffing shortages, such experiences are not a marginal phenomenon but rather symptomatic of a broader tension: The system actively recruits a diverse workforce, but offers little institutional protection against discriminatory behavior on the part of patients.

Another problem with professional identity is the paradox of self-protection. Efforts such as refusing delegated medical tasks when workload is too high or due to legal ambiguity may further weaken nurses’ structural position and reinforce the powerlessness they describe. Recent qualitative work on task shifting from physicians to nurses shows that nurses’ reluctance to assume expanded clinical responsibilities is perceived by physicians as “unwillingness to take responsibility” and can reinforce stereotypes of nurses as less responsible or less central decision makers [24]. This ambivalence demonstrates how boundary-drawing strategies that are understandable at the individual level can, at the collective level, stabilize hierarchical patterns that keep nursing voices marginal in key medical decisions in old age. The ongoing reform of nursing education in Germany aims to consolidate the previously fragmented training pathways into a single generalist profession. This reform introduces broader competencies and greater legal clarity regarding the scope of practice for nurses. In principle, it has the potential to counteract the erosion of professional identity described by the participants, by equipping nursing staff with a clearer mandate for clinical tasks and strengthening their position within interprofessional teams. However, the reform also carries risks: If structural conditions such as staff shortages and time pressures remain unchanged, expanded competencies could simply lead to additional tasks without being accompanied by a corresponding increase in autonomy, resources, or recognition. Whether the Nursing Professions Act ultimately resolves or exacerbates the professional divide observed in this study will depend crucially on how the reform is implemented at the institutional level and whether it is accompanied by meaningful structural changes.

Studies on nurses’ roles in the decision-making process for older adults with cancer have shown that, in addition to accurate geriatric assessments and provision of available information, nurses feel concerned with advocating respect for individual values and preferences of the patients [25]. The qualities of patient advocacy included protecting patients, informing them, appraising them, mediating, and supporting social justice in healthcare [26]. Our data clearly shows that nursing staff identify strongly with the task of advocacy, but struggle with asymmetries of power and voice between nurses, physicians, and relatives, which restrict their ability to translate their close knowledge of patients’ everyday lives into binding treatment decisions.

Our findings also show how staff shortage and lack of time lead to “shortcut” practices, such as using psychotropic medication to manage loneliness or PEG feeding to cope with time-intensive oral feeding. Shortcuts or unfinished nursing care are a major issue in healthcare systems of many countries that confront a growing demand for high-quality, safe care at the same or lower costs [27, 28]. A British study showed that 86% of nursing staff left one or more care activities undone due to a lack of time on their last shift. The tasks most frequently left undone were comforting or talking to patients (66%), educating patients (52%), and developing or updating nursing care plans (47%) [29]. In four post-communist countries (Croatia, the Czech Republic, Poland, and Slovakia), between 95% and 98% of nursing staff report that they are unable to perform all their duties [30]. Using shortcuts or workarounds, some nursing staff attempt to achieve the required goals despite time constraints. However, there is a risk that this time saving comes at the expense of person-centered care and dignity, as well as patient safety [31]. A systematic review of unfinished nursing care reveals a correlation with diminished job satisfaction, burnout, and intention to leave among nursing staff [27], underlining that such practices affect not only patients but also the sustainability of the nursing workforce.

Taken together, these findings indicate that nurses’ ambivalent experiences of medical progress, their moral distress and strained professional identity, and their recourse to shortcuts are closely linked expressions of the same structural conditions in contemporary elder care. From a nursing perspective, improving medical care in old age therefore requires not only better technologies and more geriatric expertise, but also reforms of financial incentives, staffing levels and decision-making structures that systematically include nursing voices. Strengthening nurses’ autonomy, advocacy role and ethical support in interprofessional teams may help to align medical interventions more closely with older people’s own ideas of a good life and a good death.

### Strengths and limitations of the study

This qualitative study offers an in-depth exploration of how nursing staff perceive the possibilities and limits of medical care in old age under current structural conditions in Germany. A key strength is the purposeful inclusion of nurses from multiple care settings—hospitals, nursing homes, and home-based nursing services—and with heterogeneous professional backgrounds, ages, and years of experience. This diversity broadens the range of experiences captured and supports the transferability of the findings to different areas of elder care within the German healthcare system. To further strengthen the trustworthiness of the study, data analysis was conducted by two researchers (EK and LM) independently, followed by joint discussion to reach consensus on categories and interpretations. In addition, the findings were discussed within the research group “Medicine, Time and the Good Life”. Nevertheless, readers should consider local contextual factors when applying these findings beyond the German healthcare system.

A notable limitation concerns the unequal distribution across care settings: while the study included nurses from hospitals, nursing homes, and home-based nursing services, hospital-based nurses were substantially overrepresented (*n* = 17), with only three participants from nursing homes. As shown in Fig. 1, we contacted 13 hospital wards, 14 nursing homes, and 20 home-based nursing services. Despite these efforts, the response rate from nursing homes was particularly low. The challenges in long-term care facilities often differ considerably from those in acute hospital wards. This imbalance may have led to a disproportionate emphasis on hospital-specific experiences and may have limited the visibility of challenges particular to nursing home care.

The use of jointly developed case vignettes on elective knee replacement and PEG insertion in very old age ensures comparability across focus groups and deliberately introduces ethically challenging scenarios that are typical of contemporary geriatric practice. The interview material supports existing theoretical frameworks such as expansion theories of morbidity, the technological imperative and moral distress. It also supports international literature on unfinished nursing care and patient advocacy.

Due to its exploratory, qualitative design, the study was based on five focus groups comprising 28 participants working Germany. Recruitment relied on a combination of purposive and convenience sampling, which may have favored the participation of nurses who were particularly engaged or burdened. While this may limit the generalizability of the findings, central issues identified—such as time pressure, workload, and resource scarcity—are widely recognized as challenges faced by nursing professionals across healthcare systems globally.

Although participants were repeatedly encouraged to contribute examples from their own professional experience—which they did—, the case vignettes may structure discussions around surgery and life-prolonging interventions for older patients. This could highlight certain forms of overtreatment and ethical conflict, while making other areas of geriatric care less visible.

Nevertheless, the focus themes and moral tensions discussed were not imposed externally but emerged directly from the participants, reflecting the nurses’ own urgent concerns. In this respect, the study gives voice to a professional group that is often underrepresented in health policy debates and underscores the need for structural change to better align medical and nursing care with patients’ lived realities and the ethical responsibilities of those who provide it.

## Conclusion

This study demonstrates that, from the perspective of nursing staff, the quality of medical care for older adults in Germany is shaped not only by medical and technological progress, but fundamentally by structural conditions including financial incentive systems, chronic staffing shortages, and restricted nursing participation in clinical decision-making. The findings underline the need for systemic reforms that strengthen nursing autonomy, provide ethical support within interprofessional teams, and align medical interventions more closely with older patients’ own values and concepts of a good life and a good death.

## Supplementary Information

Below is the link to the electronic supplementary material.

Supplementary Material 1

Supplementary Material 2

## Data Availability

The case vignettes along with the focus group guides are provided as supplementary materials. Original datasets are unavailable due to privacy regulations, though pseudonymized transcripts may be shared upon justified request from the authors.

## Abbreviations

COREQ: Consolidated Criteria for Reporting Qualitative Research
DRG: Diagnosis-Related Group
OECD: Organisation for Economic Co-operation and Development
PEG: Percutaneous Endoscopic Gastrostomy
