# Supplementary material for: Structural determinants in quality of care for older adults: a nursing perspective on medical services - qualitative findings from focus group discussions in Germany
Source: BMC Nurs. 2026 Jul 29;25:668. doi: 10.1186/s12912-026-05133-6 (PMC13418417; doi:10.1186/s12912-026-05133-6)
Supplement: Supplementary file 1 — Supplementary Material 1 [file 12912_2026_5133_MOESM1_ESM.pdf]

Appendix A: Focus Group Guide - Nursing Staff

	Narrative prompt	Checklist // Memos	Specific questions	Directive and maintaining questions
15 minutes	**Welcome**<br><br>Welcome	• Corona-Rules<br>• Audio-recording & pseudonymization<br>• Note: confidentiality<br>• Group rules<br>• Any questions?<br>• Do we need a break?<br>• Introduction round: name, motivation for participation, medical discipline		
	**Information on project**	Project is named “Medicine and the good life in old age”<br>➔ We are interested in your attitudes and experiences with medical care of older adults.<br>➔ To get closer to this topic, we have prepared two scenarios, we’d like to discuss with you.		
20 minutes	**Case vignette 1: artificial knee joint**<br><br>What do you think about this?<br><br>What advice would you give Mrs. Malsburg?	• Risks of surgery<br>• Social consequences: daughters' point of view<br>• Guilt / responsibility for her state of health<br>• Right to take risks<br>• Financial aspects: it is “worth it”?		Can you explain in more detail why you would decide like this?<br><br>What would your decision depend on?<br><br>How would you decide if … was different?<br><br>Can you put yourself in the shoes of Mrs. Malsburg’s daughters? What would then be your position?

20 minutes	**Case vignette 2: feeding via gastric tube**<br>What do you think, when you read/hear this?<br>What advice would you give Mr. Becker?	• Quality of life vs. time left<br>• Scarcity of resources (time)<br>• Consequences for family members/loved ones<br>• Risks of PEG tube		Can you explain in more detail why you would decide like this?<br>What would your decision depend on?<br>How would you decide, if … was different?<br>Would you advise your father the same?
50 minutes	**Physicians' / Nurses' own experiences**<br>I have the impression that we have collected and discussed the essential aspects of this case.<br>And now we would like to know: What experiences have you yourself had in your everyday professional life?	In your opinion/experience, what needs do seniors have?<br>What skills should be maintained to continue activities?<br>What function should medicine have?		What is the first thing that comes to your mind when you think of medical care in old age?<br>If discussion is too focused on pos./neg. aspects → specifically ask for neg./pos. examples.<br>What opportunities / problems do you think arise from medicine for older people?
10 minutes	**Closing**<br>It seems that we are slowly coming to an end... / considering the time, we should slowly come to an end.<br>So, to summarize, we could say that...	• Summary<br>• Did I forget anything?<br>• Any questions	Questions noted during discussion<br>Did I forget anything that seems important to you?<br>Do you want to share anything we didn't discuss or have not discussed in enough detail?	

Hand out form for expense allowance and certificate of attendance.
